# Genome-Wide Identification and Analysis of the Metallothionein Genes in *Oryza* Genus

**DOI:** 10.3390/ijms22179651

**Published:** 2021-09-06

**Authors:** Mingxing Cheng, Huanran Yuan, Ruihua Wang, Jianing Zou, Ting Liang, Fang Yang, Shaoqing Li

**Affiliations:** State Key Laboratory of Hybrid Rice, Hongshan Laboratory of Hubei Province, Key Laboratory for Research and Utilization of Heterosis in Indica Rice of Ministry of Agriculture, Engineering Research Center for Plant Biotechnology and Germplasm Utilization of Ministry of Education, College of Life Science, Wuhan University, Wuhan 430072, China; chengmingxing@whu.edu.cn (M.C.); huanranyuan@whu.edu.cn (H.Y.); 2020202040082@whu.edu.cn (R.W.); 201820204067@whu.edu.cn (J.Z.); 2015202040066@whu.edu.cn (T.L.); fang-yang@whu.edu.cn (F.Y.)

**Keywords:** *Oryza sativa*, metallothionein (MT), phylogenetic analysis, promoter activity, expression analysis, subcellular localization

## Abstract

Metallothionein (MT) proteins are low molecular mass, cysteine-rich, and metal-binding proteins that play an important role in maintaining metal homeostasis and stress response. However, the evolutionary relationships and functional differentiation of *MT* in the *Oryza* genus remain unclear. Here we identified 53 *MT* genes from six *Oryza* genera, including *O. sativa* ssp. *japonica*, *O. rufipogon*, *O. sativa* ssp. *indica*, *O. nivara*, *O. glumaepatula*, and *O. barthii*. The *MT* genes were clustered into four groups based on phylogenetic analysis. *MT* genes are unevenly distributed on chromosomes; almost half of the *MT* genes were clustered on chromosome 12, which may result from a fragment duplication containing the *MT* genes on chromosome 12. Five pairs of segmental duplication events and ten pairs of tandem duplication events were found in the rice *MT* family. The Ka/Ks values of the fifteen duplicated *MT* genes indicated that the duplicated *MT* genes were under a strong negative selection during evolution. Next, combining the promoter activity assay with gene expression analysis revealed different expression patterns of *MT* genes. In addition, the expression of *OsMT* genes was induced under different stresses, including NaCl, CdCl_2_, ABA, and MeJ treatments. Additionally, we found that *OsMT* genes were mainly located in chloroplasts. These results imply that *OsMT* genes play different roles in response to these stresses. All results provide important insights into the evolution of the *MT* gene family in the *Oryza* genus, and will be helpful to further study the function of *MT* genes.

## 1. Introduction

Metallothioneins (MTs) are a family of low molecular mass (4–8 kD), cysteine (Cys)-rich proteins that bind metals via thiol groups of cysteine (Cys) residues [1]. *MT* genes are widespread in prokaryotes, plants, and animals [2]. MTs have a strong affinity for both essential (zinc, copper, selenium) and xenobiotic (cadmium, lead, mercury) metals, binding them through specific Cys-Cys and Cys-Xxx-Cys motifs [3,4,5]. As a result, MTs have various biological functions, including protective effects, resisting metal toxicity, controlling oxidative stress, and regulating physiological homeostasis [6,7,8].

So far, there is increasing evidence that various abiotic stresses can regulate *MT* gene expression; for example, drought, abscisic acid, salt, environment temperature, and reactive oxygen species [9,10,11,12]. This shows the importance of plant *MTs* genes in response to abiotic stress. For example, an *MT-2* gene (*MT2*) was upregulated in boron-stressed tomato plants, implying that it may protect against boron stress [13]. In *Chloris virgata* Swartz (*C. virgata*), *ChlMT1* expression was induced by several abiotic stresses, such as salts (NaCl and NaHCO_3_), ROS inducer (paraquat), and metals (CuSO_4_, Z nSO_4_, and CdCl_2_). Interestingly, alien *ChlMT1* overexpression could significantly improve the tolerance of yeasts to reactive oxygen species and salinity [14]. Similarly, the ectopic expression of *OsMT1e-P* can increase tolerance toward multiple abiotic stresses in transgenic tobacco; also, transgenic plants could survive and produce viable seeds under salt stress [15].

In many higher plant species, *MT* genes have been reported to be expressed specifically in different tissues. For example, the expression level of *OsMT* genes in mature rice plants is extremely high in stems relative to leaf blades, leaf sheaths, endosperm, and roots [16]. *METALLOTHIONEIN2b* (*OsMT2b*) is preferentially expressed in immature rice panicles, the scutellum of germinating embryos, and the primordium of lateral roots [2]. In cucumbers, *CsMT* genes exhibit different tissue expression patterns [17].

Although some *MT* genes have been characterized in rice [18], *Arabidopsis* [19], cucumbers [17], tomatoes [20], and soybeans [21], systematic and thorough studies are lacking in plants, especially in the *Gramineae* species. Most of the *MT* family remains unclear to date, limiting the depth of understanding of the evolutionary patterns of MTs in *Gramineae*. Therefore, it would be of important to study their evolution systematically and the possible physiological role of the *MT* gene family in *Gramineae*.

For this purpose, here, we identified and systematically characterized the structure, distribution, and evolution of the *MT* genes in the five *Oryza* AA genome species, including *O. sativa*, *O. rufipogon*, *O. nivara*, *O. glumaepatula*, and *O. barthii*. This will deepen our understanding and facilitate further investigation of the function of MTs in *Oryza* species.

## 2. Results

### 2.1. Identification and Structural Characterization of the MT Genes in the Oryza Genus

**Figure 1:**
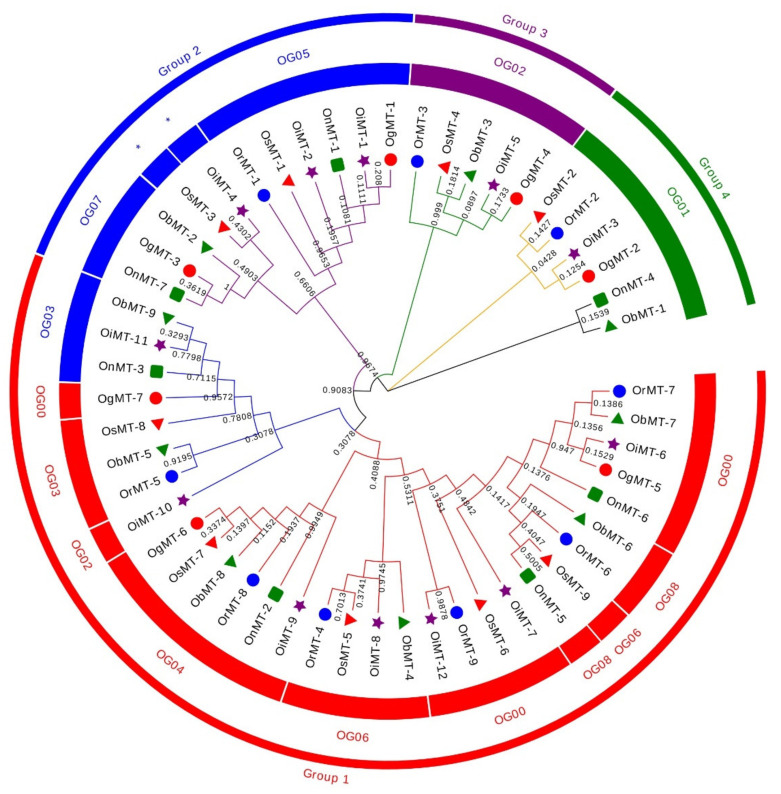
Phylogenetic tree of *MT* genes based on protein sequences from six *Oryza* species/subspecies. ClustalW is used for multiple sequence alignment. MEGA v.7.0 is adopted for phylogenetic reconstruction using the neighbor-joining (NJ) clustering method. Bootstrap numbers (1000 replicates) are shown. Different color of circles represents different subfamilies. Different-shaped markers indicate the different species. The numbers inside the red circles represent the different orthogroups (OGs). * means unassigned proteins.

The hidden Markov model searching for proteins containing a metallothionein domain (Pfam accession no. PF01439) was downloaded from Pfam (http://pfam.xfam.org/) [22]. There were 9, 9, 12, 7, 7, and 9 *MT* members identified in *O. sativa* ssp.*japonica*, *O. rufipogon*, *O. sativa* ssp. *indica*, *O. nivara*, *O. glumaepatula*, and *O. barthii*, respectively. These genes are mainly dispersed across nine chromosomes, with a bias to Chr 12, where more than half of the genes are located on this chromosome (Supplementary Table S1). The 53 *MT* genes can be classified into four subfamilies according to the sequence identity. In detail, Chr1, Chr3, and Chr11 had six, six, and five genes, respectively, whereas two genes were found on Chr2, Chr5, and Chr8, and three genes on Chr10 (Supplementary Figure S1). Interestingly, the genes on Chr2, Chr8, and Chr12 were grouped into group 1; the Chr1, Chr5, and Chr10 *MT* genes belonged to group 2; the *MT* genes on Chr11 belonged to group 3; and the *MT* genes on Chr3 belonged to group 4 (Figure 1 and Supplementary Table S1). This characteristic is consistent with the classification of *MT* genes, reflecting the strict conservation of *MT* genes among the six *Oryza* species/subspecies.

**Figure 2:**
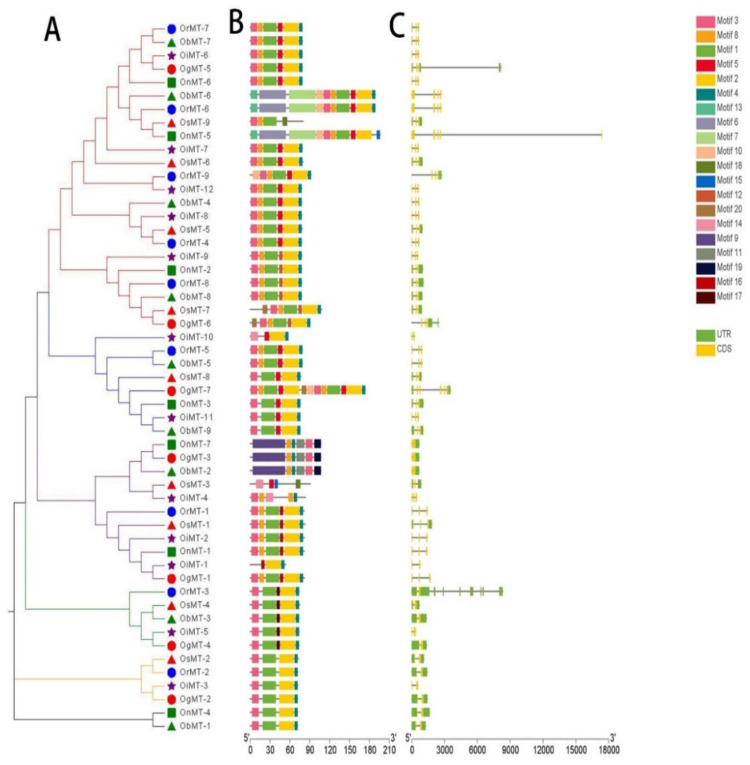
The phylogenetic tree (**A**), motif composition (**B**), and exon/intron structure (**C**) of the *MT* genes in six *Oryza* species/subspecies. (**A**) Sequence alignments and the NJ-phylogenetic trees were made using ClustalW and MEGA v.7.0, respectively. A bootstrap number (1000 replicates) is adopted; (**B**,**C**) the widths of the gray bars represent the relative lengths of genes and proteins. The green boxes and gray lines display exons and introns, respectively.

The phylogenetic tree of *MT* genes based on protein sequences from six *Oryza* species/subspecies is shown in Figure 2A. Furthermore, the *MT* gene’s structure was characterized in order to acquire more viewpoints into the structural diversity of *MT* genes in the *Oryza* species (Figure 2C). Results indicated that the intron number of *MT* genes in six *Oryza* species/subspecies ranged from 1 to 8, and the exon number ranged from 2 to 6. A total of 31 *MT* genes (58.5%) had three exons, followed by the 16 (30.2%), 4 (7.5%), 1 (1.9%), and 1 (1.9%) gene, possessing two, four, five, and six exons, respectively. Further, 20 conserved motifs were identified from the 53 *MT* proteins using the MEME [23] (Supplementary Table S2), and all 53 *MT* proteins showed a similar motif arrangement (Figure 2B). Notably, we found that MTs from the same group showed variations in the number and length of exons/introns, suggesting the functional diversification of the *MT* genes in the same group.

### 2.2. Chromosomal Distribution, and Evolutionary Characters

**Figure 3:**
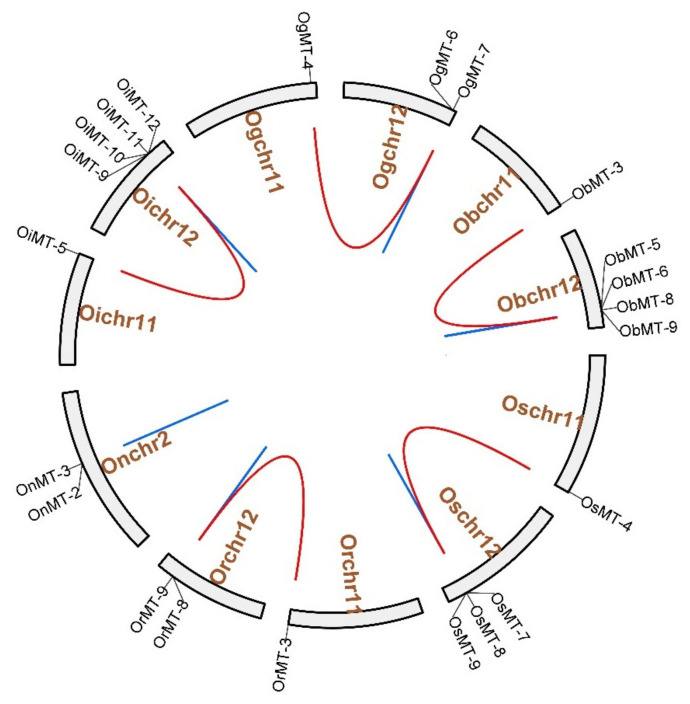
The chromosome location and duplication events of *MT* genes in six species/subspecies. Os represents *Oryza sativa* ssp. *japonica*. Or represents *Oryza rufipogon*. On represents *Oryza nivara*. Oi represents *Oryza sativa* ssp. *indica*. Og represents *Oryza glumaepatula*. Ob represents *Oryza barthii*. The location of each *MT* gene is marked with a gray line using Circos software. The whole-genome duplication (WGD) or segmental duplication/tandem duplication gene pairs are linked by red/blue lines.

The chromosome location results showed that *MT* genes show an unbalanced distribution pattern, where no *MT* genes were mapped on Chr4, Chr6, Chr7, and Chr9 (Figure 3 and Supplementary Figure S1). Furthermore, we discovered five pairs of segmental duplication events and ten pairs of tandem duplication events in the six *Oryza* species/subspecies (Table 1 and Figure 3). Interestingly, segmental duplication events were detected in all species/subspecies except *Oryza nivara* (Figure 3); moreover, nine pairs of tandem duplication clusters were observed on chromosome 12, reflecting that gene duplication may be the major cause for the expansion of the *MT* family in the *Oryza* species. The segmental duplication events of these six gene pairs were estimated to occur between 7.65 and 10.32 Mya (Table 1).

Next, Ka/Ks values of *MT* duplicate gene pairs were calculated to evaluate the driving force underlying *MT* gene evolution. The results showed that Ka/Ks values of the 15 duplicated *MT* genes ranged from 0.1318 to 0.7686, and all Ka/Ks values were less than 1 (Table 1), indicating that the duplicated *MT* genes were under a strong negative selection during evolution [24].

To better understand the evolutionary relationship of the *MT* genes in the *Oryza* genus, the orthogroup clustering was analyzed and a phylogenetic tree was constructed, and showed nine orthogroups in six *Oryza* genera; namely, eight in *O. sativa* ssp. *japonica*, eight in *O. rufipogon*, seven in *O. sativa* ssp. *indica*, eleven in *O. nivara*, eight in *O. glumaepatula*, and nine in *O. barthii*. However, *OsMT-3* and *OiMT-4* were assigned to the *MT* family (Supplementary Table S3 and Supplementary Table S4). Furthermore, the gene numbers in each orthogroup were different, ranging from 3 to 10. Orthogroup 0 was the largest, and single-copy orthogroups were found in orthogroups 1 and 4. Besides, we found that the number of orthologs was also different among these species (Table 1). These results showed that the unequal loss and expansion of most orthogroups might have occurred during the domestication process.

### 2.3. Collinearity Relations of O. sativa ssp. japonica with Other Tested Species

To evaluate the evolutionary relationship of the *MT* genes within *Gramineae*, the molecular phylogeny of the *MT* family was analyzed using the MCScanX toolkit. Referenced to the genome of *japonica* rice Nipponbare, we found seven, five, nine, six, and seven collinear gene pairs between *O. sativa* ssp.* japonica* and *O. rufipogon*, *Oryza nivara*, *Oryza sativa* ssp. *indica*, *Oryza glumaepatula*, and *Oryza barthii*, respectively (Supplementary Figure S2). The *MT* genes showed a strongly conserved collinearity among the six *Oryza* species and subspecies, and the collinearity of *MT* genes between *japonica* and *indica* was closer than the other species, supporting their close evolutionary distance.

### 2.4. Promoter Activity and Cis-Elements Identification of OsMT Genes in O. sativa ssp. japonica

Cis-elements in the promoter usually play a vital role in responding to different environments and determining the tissue-specificity of genes [25,26]. Thus, potential cis-regulatory elements in the promoter regions of *japonica* rice *OsMT* genes were identified by searching the PlantCARE database [27]. In total, 31 types of cis-regulatory elements were identified (Supplementary Figure S3); they can be primarily classified into three categories based on their functionality: phytohormone response, growth and development, and stress response [28]. The cis-regulatory elements in the growth and development category had a higher percentage than the other two categories (Supplementary Figure S3A,B). In the growth and development category, the light-responsive/responsiveness subcategory had a total of 74 motifs that belonged to 15 types of cis-elements (Supplementary Figure S3A), which indicated that light-responsive/responsiveness is widely present in the promoter region of *MT* genes. In the phytohormone response category, the MeJA-responsiveness element was the largest subcategory (40), which includes the TGACG- and CGTCA motif, followed by the abscisic acid responsiveness subcategory, including the ABRE cis-element (Supplementary Figure S3A). The top three subcategories in the stress response category were the anaerobic induction, drought-inducibility, and low-temperature responsiveness elements (Supplementary Figure S3A). The marked cis-element related to biological and abiotic stress in the promoters means that *OsMT* genes are widely involved in the environmental stress response.

Further analysis showed that cis-regulatory elements are unevenly distributed in the *OsMT* genes, and that some cis-regulatory elements were preferentially present on individual *OsMT* genes. For example, *OsMT-5* and *OsMT-8* had many MeJA-responsive cis-regulatory elements, and *OsMT-7* had the most auxin regulatory elements (Supplementary Figure S3C), which is the functional specificity for a few of the *MT* genes.

**Figure 4:**
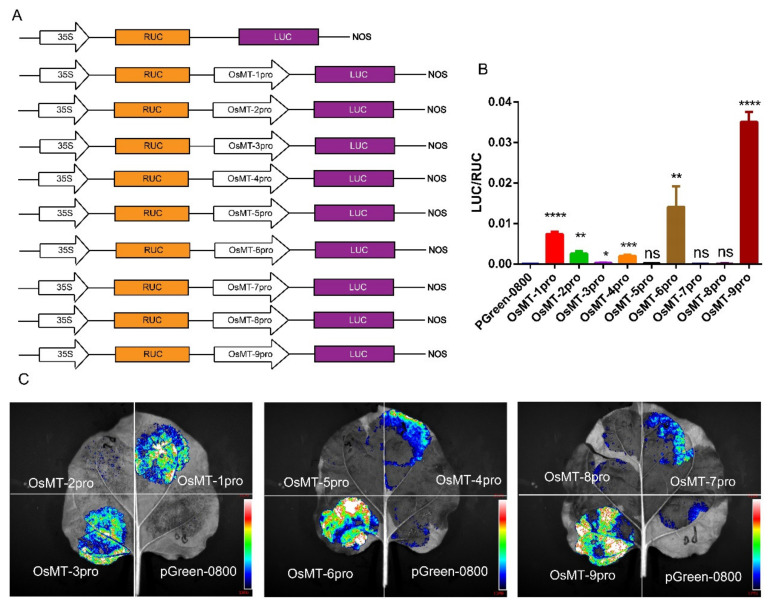
Analysis of the promoter function of *OsMT* genes in vivo: (**A**) the vector constructs are used in the dual-luciferase assay; (**B**) dual-luciferase assay in the protoplast of rice; (**C**) dual-luciferase assay in *Nicotiana benthamiana* leaves. The error bars show the standard deviations of the three independent biological replicates. Significance analysis was performed using *t*-test; *: *p* < 0.05, **: *p* < 0.01, ***: *p* < 0.001, ****: *p* < 0.0001.

The functional specificities of plant genes are often reflected by the promoter activities [29]; thus, the promoter activity of the nine *OsMT* genes in rice was investigated in planta using pGreen-0800 as a control, where the vector constructs are used in the dual-luciferase assay (Figure 4A). Results showed that the *OsMT-9* promoter showed the highest LUC/RUC ratio in the protoplast of rice (Figure 4B), the promoter of *OsMT-5*, *OsMT-7*, and *OsMT-8* showed the weakest activity, and the promoter of *OsMT-1, OsMT-3, OsMT-4*, *OsMT-6*, and *OsMT-7* showed a higher fluorescence intensity than the control in *Nicotiana benthamiana* leaves (Figure 4C), reflecting a tissues-specific functional differentiation of the *OsMT* genes in rice.

### 2.5. Expression Profiling of OsMT Genes in Rice

**Figure 5:**
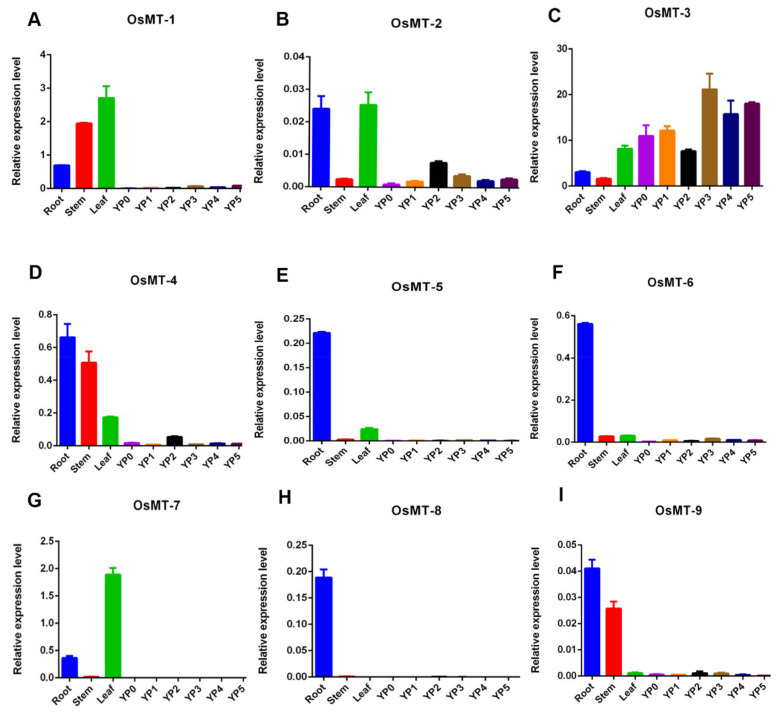
Expression profiles of *OsMT* genes in different tissues. Real-time quantitative PCR, YP0: 0–0.5 cm panicle, YP1: 0.5–1 cm panicle, YP3: 1–2 cm panicle, YP4: 2–3 cm panicle, YP5: 3–4 cm panicle. The error bars show the standard deviations of the three independent qRT-PCR biological replicates. (**A**–**I**) The expression profiles of 9 *OsMTs* in different tissues.

The expression profile analysis of a single gene family may provide important clues for the functional differentiation [30]. For this purpose, expression profiles of *OsMT* genes were assayed in rice. Results showed that almost all of the *OsMTs* were preferentially expressed in the vegetative tissues, particularly in roots, revealing the functional similarity of *OsMT-3* genes in rice (Figure 5). Exceptionally, *OsMT-3* was highly expressed in all vegetative and productive tissues, especially in young inflorescence, indicating the functional differentiation of *OsMT-3* from the others in rice.

### 2.6. Expression Patterns of MT Genes in Rice Roots and Shoots under Various Abiotic Stresses

**Figure 6:**
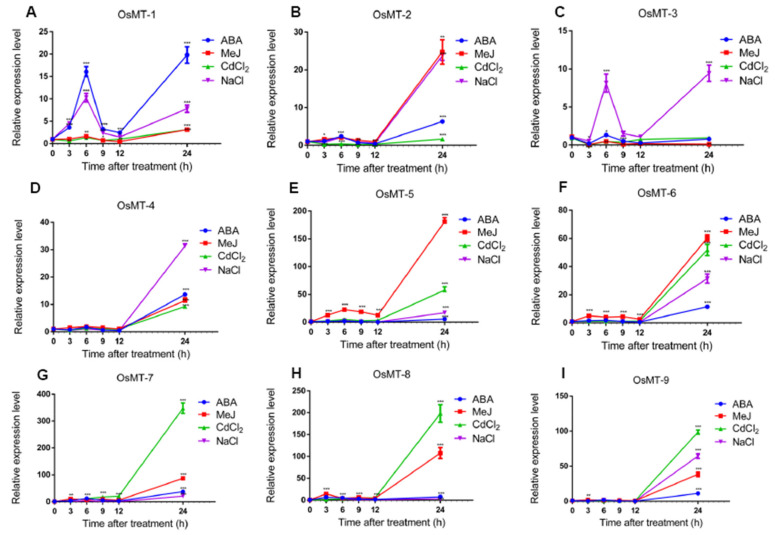
qRT-PCR of relative expression of *OsMT* genes in roots at the rice seedling stage under various abiotic stress treatments, including NaCl, CdCl_2_, ABA, and MeJ: (**A**–**I**) the expression of *OsMT* genes was calculated at 3, 6, 9, 12, and 24 h of treatments compared with the expression value at 0 h, which was normalized to 1. The error bars show the standard deviations of the three independent qRT-PCR biological replicates. Significance analysis was performed using *t*-test; *: *p* < 0.05, **: *p* < 0.01, ***: *p* < 0.001.

The *OsMTs* promoter cis-element analysis indicated that MTs promoters harbor an amount of ABA, JA, and SA response-related motifs, meaning that the *MT* genes in rice may be involved in the stress response. Therefore, we investigated the expression of rice *MT*s under four chemical treatments, including NaCl, CdCl_2_, ABA, and MeJ, to mimic environmental stresses. Results showed that all of the *OsMT* genes in roots had a similar response pattern at a transcriptional level under the four treatments. Apart from *OsMT-1* and *OsMT-3*, which had two apparent expression peaks at 6 h and 24 h after treatment, the other seven *OsMT* genes all showed a steep increase trend from the 12 h to 24 h after different treatments. Relatively, the *OsMT-1* was most sensitive to ABA, *OsMT-2* was most sensitive to NaCl and MeJ, *OsMT-3* and *OsMT-4* was most sensitive to NaCl, *OsMT-5* and *OsMT-6* was most sensitive to MeJ, and *OsMT-7*, *OsMT-8*, and *OsMT-9* was most sensitive to CdCl_2_ (Figure 6), reflecting the functional differentiation among *OsMTs* to some extent in rice.

**Figure 7:**
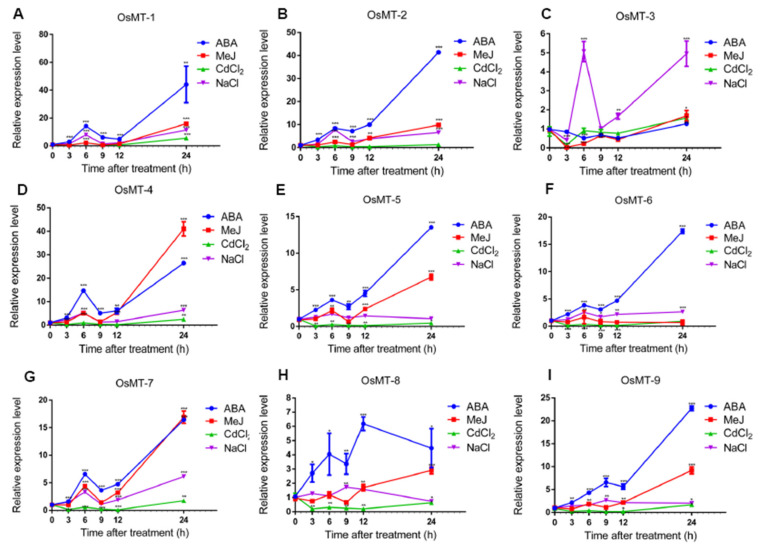
qRT-PCR of relative expression of *OsMT* genes in shoots at the rice seedling stage under various abiotic stress treatments, including NaCl, CdCl_2_, ABA, and MeJ: (**A**–**I**) the expression of *OsMT* genes was calculated at 3, 6, 9, 12, and 24 h of treatments compared with the expression value at 0 h, which was normalized to 1. The error bars show the standard deviations of the three independent qRT-PCR biological replicates. The significance analysis was performed using *t*-test; *: *p* < 0.05, **: *p* < 0.01, ***: *p* < 0.001.

In order to compare the difference in expression patterns of the *OsMT* genes between shoots and roots under abiotic stresses, the expression of *OsMT* genes in shoots were further investigated under treatments of NaCl, CdCl_2_, ABA, and MeJ (Figure 7). Results indicated that, with the exception *OsMT-8*, all of the other *OsMT* genes in shoots have a similar expressional trend as that in roots under various abiotic stresses; the *OsMTs* in shoots seem to respond more rapidly than those in roots, but the response intensity of *OsMTs* in shoots is apparently lighter than that in roots. The *OsMTs* expression was upregulated at all time points, and the expression accelerated steeply at 12 h under abiotic stresses, implying the consistency of the regulation of *OsMTs* between roots and shoots in rice under abiotic stresses of NaCl, CdCl_2_, ABA, and MeJ. Unexpectedly, the expression patterns of *OsMT-8* showed a more complicated pattern than the others; the *OsMT-8* expression changed in a different and even opposite way under the four chemical treatments. This means that the *OsMT-8* expression in shoots is possibly regulated by some factors that are absent in roots.

### 2.7. Subcellular Localization of OsMT Genes

**Figure 8:**
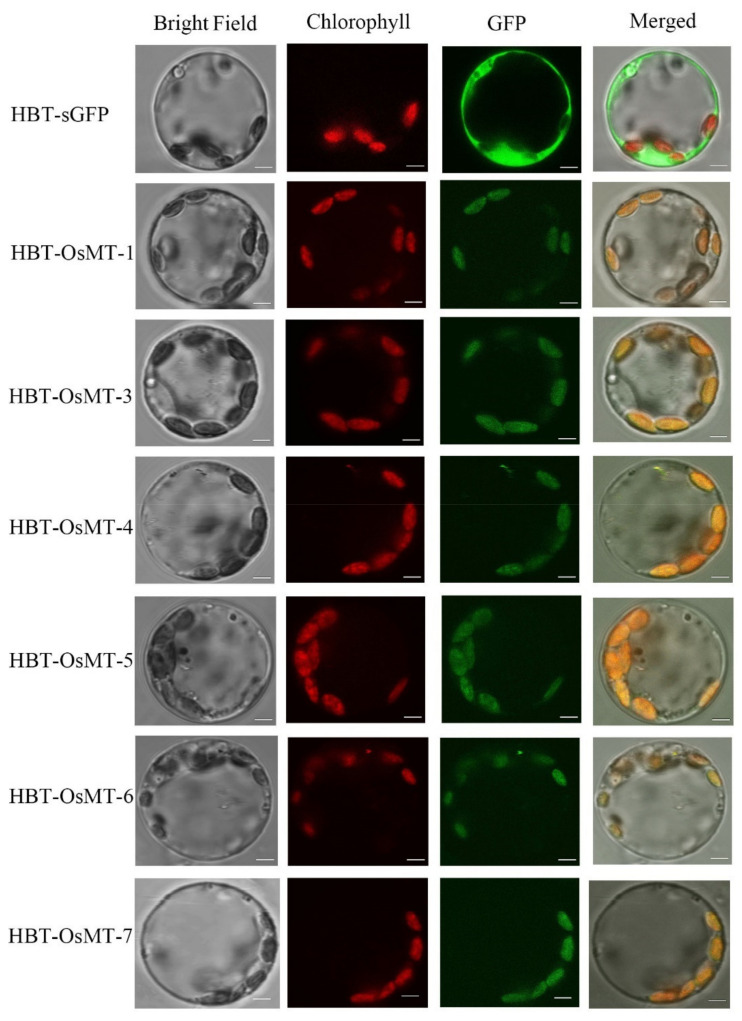
Subcellular localization of OsMT proteins in rice green seedling protoplasts. The empty vector, HBT-sGFP, was used as a control. Bright-field images (Bright Field), chlorophyll autofluorescence (Chlorophyll), fluorescence of eGFP fusion protein (GFP), and merged images (Merged) were examined using laser scanning confocal microscopy. Bars = 3 μm.

All of the subcellular localizations of the *OsMT* genes were predicted in the chloroplast using WoLF PSORT (https://wolfpsort.hgc.jp/). OsMT-GFP fusion proteins were transiently expressed in rice protoplasts, except *OsMT-2*, *OsMT-8*, and *OsMT-9*, due to no full-length coding sequence being obtained. Results showed that, unlike the GFP signal of the empty vector mainly detected in the cytoplasm and plasma membrane, the GFP signals of the OsMT-GFP fusion proteins were completely confined to chloroplasts in cells (Figure 8), indicating that these OsMTs are mainly located in and serve a function in chloroplasts.

## 3. Discussion

With the rapid development of genome sequencing technology, more and more high-quality reference genomes of plants are accessible, which makes the systematic study of the structure and function of *MT* family genes feasible. In most dicotyledons, the number of *MT* genes is usually fewer than 20 members, such as Arabidopsis [19], cucumbers [17], tomatoes [20], and soybeans [21], which each have 4, 3, 4, and 9 *MT* genes. In this study, we found the number of *MT* genes ranged from seven to twelve in the *Oryza* AA genome species (Figure 1 and Table 1), and showed a high sequence and structural identity among different species, reflecting the evolutionarily conserved collinear relations of the *MT* genes in the *Oryza* AA genome species.

Previous studies have revealed that the expression of metallothionein genes is induced by diverse abiotic stresses and a variety of environmental stimuli in response to plant growth and development [8,9,10,13,15,17,31]. Our results showed that, although the expression profiles have some differences among *OsMT* genes in rice, and even *OsMT-3* shows a tissue-specific expression pattern, they all showed an extremely high expression level in vegetative tissues, particularly in roots and leaves or stems (Figure 5), highlighting their functional specificity related to roots. In agreement with this character is that, when we treated the rice seedling with NaCl and CdCl_2_, the *OsMT* genes responded swiftly within 12 h and accumulated greatly in roots and shoots (Figure 6 and Figure 7), revealing the important function of *MT* genes involved in the resistance of plants to environmental stresses. This is consistent with the previous reports that some *MT* genes exhibited a higher metal tolerance, particularly for Cd^2+^ [10,32,33].

Usually, the cis-elements’ response to ABA, JA, and SA in the promoter is the hallmark of a gene involved in abiotic/biotic stress environments, because ABA, JA, and SA are the three central hormones in plants that integrate the critic genes and pathways to respond to the challenge of side environments. Noticeably, in this study, many cis-elements related to stress and hormone responses were detected in the promoter regions of each *OsMT* gene (Supplementary Table S5), further hinting that *MT* genes are probably involved in the anti-stress response of plants. Strikingly, when we treated the rice seedling with ABA or MeJ, the rice roots and shoots showed almost completely similar expression patterns as those of rice treated with NaCl and CdCl_2_ (Figure 6), meaning that the *OsMT* genes of rice are commonly induced by NaCl, CdCl_2_, ABA, and MeJ. It is worth mentioning that the *OsMT* proteins are all located in the chloroplast, which is in agreement with the concept that chloroplasts are organelles for ABA, SA, and MeJ synthesis [34,35,36,37], and are strongly associated with the stress response during plant growth and development [38,39,40].

Gene expansion will help to broaden the function of a gene family so as to better adapt the environment of an organism. Here, fifteen duplication events were detected in the *MT* family of six *Oryza* species/subspecies, including five pairs of segmental duplication events and ten pairs of tandem duplication events (Figure 3). Interestingly, *O. sativa* ssp.* indica* has the most duplication events (Table 1). As we know, the *indica* rice has been planted widely in various environments in the five continents, which are Asia, Europe, Africa, Australia, and America, meaning that it received a stronger selection than the other four *Oryza* species and *japonica* subspecies in the last thousand years. The *MT* family is tightly related to environment stress response. We deduce that a strong human selection may promote the expansion and accumulation of the *MT* family so as to well adapt to various environments around the world [30].

## 4. Conclusions

This study identified 53 *MT* genes from six *Oryza* species/subspecies, including *O. sativa* ssp.* japonica*, *O. rufipogon*, *O. sativa* ssp.* indica*, *O. nivara*, *O. glumaepatula*, and *O. barthii*. The Ka/Ks values of the fifteen duplicated *MT* genes indicated that the *MT* genes were under a strong negative selection. Duplication led to various expression patterns and the functional differentiation of the *MT* genes, so as to adapt different environment stresses, as shown in the treatments of NaCl, CdCl_2_, ABA, and MeJ in rice. Conclusively, this work partly uncovers the potential roles of *OsMT* genes that are played in response to environment stresses, which may provide a reference for the functional analysis of the *MT* genes in the other *Gramineae* species in the future.

## 5. Materials and Methods

### 5.1. Identification and Phylogenetic Tree Construction of MT Genes

To identify *MT* genes in *Oryza* genus, the whole-genome data of six representative *Oryza* species: *O. barthii*, *O. indica*, *O. glumipatula*, *O.nivara*, *O. rufipogon*, and *O. sativa japonica*, were downloaded from Ensembl Plants release 41 [41] and Phytozome v.12 [42]. In addition, PF01439 was downloaded from Pfam (http://pfam.xfam.org/, accessed on 10 December 2020). Furthermore, all candidate proteins were separately identified by HMMER v.3.2.1 [43] and BLASTP [44]. Finally, SMART (http://smart.embl-heidelberg.de, accessed on 10 December 2020) and Pfam (http://pfam.xfam.org/search/sequence, accessed on 10 December 2020) were used to verify these sequences [45,46].

Multiple sequence alignment of full-length *MT* protein sequences was performed by ClustalW [47], and an unrooted phylogenetic relationship was constructed using MEGA7 [48] using the neighbor-joining (NJ) method with the Jones–Taylor–Thornton (JTT) model based on 1000 bootstrap replicates.

### 5.2. Gene Structure, Conserved Motifs, and Phylogenetic Analysis

Exon/intron site and length data were extracted based on six respective genome annotation GFF files from Ensembl Plants [41]. The software MEME Suite v.5.3.3 [23] was used to identify conserved motifs with a maximum number of 20. The phylogenetic tree was drawn by EvolView v.3 [49], and exon/intron structures were shown using TBtools v.1.0971 proportionally [50].

### 5.3. Chromosomal Locations, Gene Duplication Analysis, and Orthogroup Analysis

The collinearity relationships were obtained using BLAST search with default parameters and generated using a procedure in ColinearScan using the MCScanX toolkit [51]. All *Oryza MT* genes were classified into various types of duplications. First, a schematic of the putative duplications of the *MT* genes was constructed using the Circos software [52]. Then, the putative WGDs/segmental duplications of *MT* genes were connected by links. Finally, the synonymous (Ks) and nonsynonymous (Ka) substitution rates were estimated using DnaSP v.6.0 (http://www.ub.edu/dnasp/) [53]. The divergence time (T) was estimated by T = Ks/(2 × 9.1 × 10^−9^) × 10^−6^ million years ago (MYa) [46].

The orthogroup was identified using OrthoFinder v.2.5.4 with a cut-off e-value of 1 × 10^−3^ [54]. Then, STAG and STRID algorithms were used to rebuild the phylogenetic tree of the selected species based on the detected orthogroup.

### 5.4. Quantitative RT-PCR (qRT-PCR) Analysis of MT Genes in O. sativa japonica

All RNA extraction and reverse transcription were performed by TRIzol Reagent (Invitrogen, Carlsbad, CA, USA) according to the manual. First-strand cDNA was synthesized from 2 µg DNase-treated RNA using HiScript III 1st Strand cDNA Synthesis Kit (+gDNA wiper) (Vazyme, Nanjing, China). Hieff qPCR SYBR Green Master Mix (No Rox) (Yeasen, Shanghai, China) was used for the qRT-PCR. The actin gene was used as an internal control. The relative expression levels of MST genes were calculated using the 2^−∆∆CT^ method (Supplementary Table S6).

### 5.5. Plant Material and Treatments

The rice cultivar ZH11 (*O. sativa japonica*) was used in this study for expression analysis of *MT* genes under different biological stresses, including NaCl, CdCl_2_, ABA, and MeJ treatments at different time points. All seedlings were grown in a growth chamber with the climatic conditions set at 28 °C, 14 h light, and 10 h dark, with 60% relative humidity for 12 d.

For different biological stress treatments, the roots of 12-day-old seedlings were washed, followed by immediate transfer into abscisic acid solution (ABA, 100 µM), methyl jasmonate solution (MeJ, 100 µM), CdCl_2_ (200 µM), and NaCl solution (200 mM), respectively. Roots and shoots were sampled at 0, 3, 6, 9, 12, and 24 h during the light period after applying treatments. Three biological replicates were produced for every treatment, collected from 12 seedlings, and pooled together. The samples were immediately frozen with liquid nitrogen and stored at −80 °C for further use.

### 5.6. Subcellular Localization of OsMT Genes

The full-length coding sequences of OsMT were amplificated using gene-specific primers and then inserted behind the cauliflower mosaic virus CaMV 35S promoter into the HBT-GFP vector to produce a structure that is 35S: OsMT: GFP fusion vector. OsMT-GFP fusion proteins were transiently expressed in rice protoplasts. The empty HBT vector was used as a control. Protoplasts were prepared using the rice seedlings of ZH11 plants. Briefly, for the protoplast transformation, 20 µL OsMT:GFP was mixed with 200 µL protoplasts and 240 µL PEG solution (40% PEG4000, 0.6-M mannitol, and 100-mM CaCl_2_) for 15 min. After washing twice with W5 solution (154 mM NaCl, 125 mM CaCl_2_, 5 mM KCl, 5 mM glucose, 2 mM MES, pH 5.7 by KOH), the protoplasts were cultured at 28 °C overnight. The protoplasts were observed for GFP and RFP signals using the FV1000 confocal system. The primers used for subcellular localization are listed in Supplementary Table S7.

### 5.7. Dual-Luciferase Assays

To investigate the promoter of *OsMT* genes, the promoter sequence of *OsMT* genes was amplified by PCR from the ZH11 genomic DNA and constructed into pGreenII 0800-LUC reporter vectors in front of the luciferase (LUC) gene. Besides, the renilla luciferase (REN) reporter gene was driven by the CaMV 35S promoter as a control in each transformation. The different reporters were transformed into rice protoplasts according to a previous method [55]. The luciferase activities were measured using the Dual-Luciferase Reporter Assay System (Promega, Madison, WI, USA) and compared with empty vector pGreenII 0800-LUC. The relative luciferase activity was calculated by the ratio of firefly luciferase and renilla luciferase (fLUC/rLUC). Using GV3101(pSoup-p19) *Agrobacterium*-mediated transformation, the reporter was injected into 30-day *N. benthamiana* leaves. The injected *N. benthamiana* was grown in a greenhouse for three days. Finally, luciferin was injected into the leaves to test luciferase intensity in Tianeng 4800 automatic chemiluminescence image analysis system. The primers used for dual-luciferase assays are listed in Supplementary Table S8.

## Figures and Tables

**Table 1:** Ka, Ks, and Ka/Ks values for duplicated gene pairs in rice.

Seq1	Seq2	Ks	Ka	Ka/Ks Ratio	Date (MY)	Duplication Type
OsMT-4	OsMT-7	0.1392	0.9012	0.1544	7.65	WGD or segmental duplications
OsMT-7	OsMT-8	0.1217	0.7707	0.1579	6.69	tandem duplication
OsMT-8	OsMT-9	0.2080	0.6090	0.3416	11.43	tandem duplication
OrMT-3	OrMT-8	0.1392	0.9745	0.1428	7.65	WGD or segmental duplications
OrMT-8	OrMT-9	0.0882	0.4947	0.1784	4.85	tandem duplication
OnMT-2	OnMT-3	0.1117	0.7852	0.1422	6.14	tandem duplication
OiMT-5	OiMT-9	0.1392	0.9745	0.1428	7.65	WGD or segmental duplications
OiMT-9	OiMT-10	0.2444	0.6186	0.3951	13.43	tandem duplication
OiMT-10	OiMT-11	0.2195	0.2856	0.7686	12.06	tandem duplication
OiMT-11	OiMT-12	0.1101	0.4226	0.2606	6.05	tandem duplication
OgMT-4	OgMT-6	0.1878	0.9203	0.2041	10.32	WGD or segmental duplications
OgMT-6	OgMT-7	0.1861	0.5980	0.3112	10.22	tandem duplication
ObMT-3	ObMT-8	0.1392	1.0556	0.1318	7.65	WGD or segmental duplications
ObMT-5	ObMT-6	0.1212	0.4932	0.2458	6.66	tandem duplication
ObMT-8	ObMT-9	0.1117	0.7852	0.1422	6.14	tandem duplication

Synonymous (Ks) and nonsynonymous (Ka) substitution rates of duplicate gene pairs (Ka/Ks ratios).

## Data Availability

Data is contained in Supplementary Materials.

## Supplementary Materials

The following are available online at www.mdpi.com/article/10.3390/ijms22179651/s1.

## Abbreviations

MT	Metallothionein
Os	Oryza sativa ssp. japonica.
Or	Oryza rufipogon.
On	Oryza nivara.
Oi	Oryza sativa ssp. indica.
Og	Oryza glumaepatula.
Ob	Oryza barthii.
ABA	Abscisic acid
MeJ	Methyl Jasmonate
qRT-PCR	Quantitative real-time PCR
