# Supplementary material for: Genome-Wide Identification and Analysis of the Metallothionein Genes in Oryza Genus
Source: Int J Mol Sci. 2021 Sep 6;22(17):9651. doi: 10.3390/ijms22179651 (PMC8431808; doi:10.3390/ijms22179651)
Supplement: Supplementary file 1 [file ijms-22-09651-s001.zip › Supplementary/Supplementary Figures.pdf]

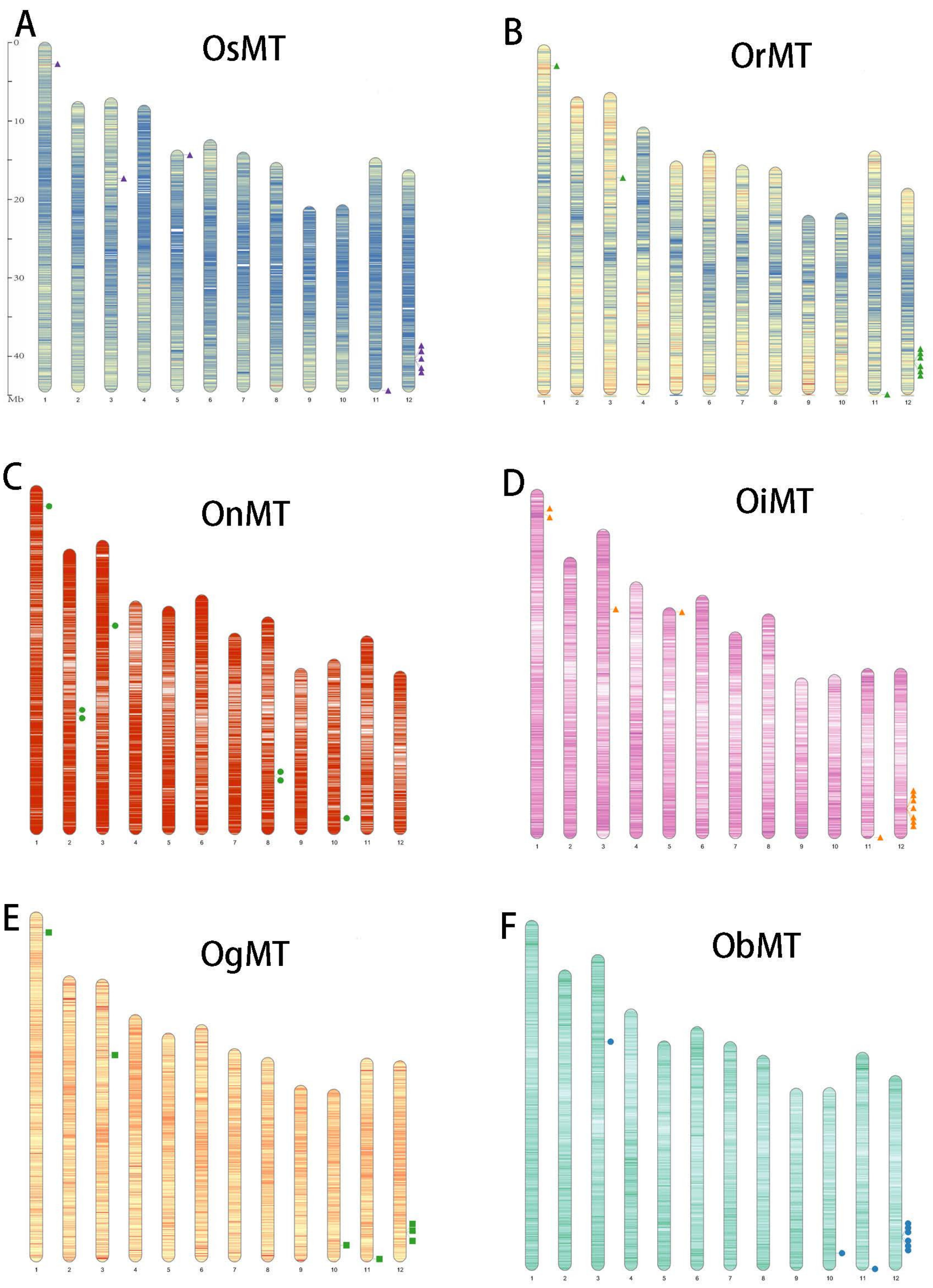

Supplementary Figure S1. (A-F) Chromosome mapping of *MT* genes in the six *Oryza* genomes. Chromosome numbers are indicated at the bottom of each bar. Scale is represented in mega bases (Mb) and lines in the background chromosome indicate all genes within the genomes.

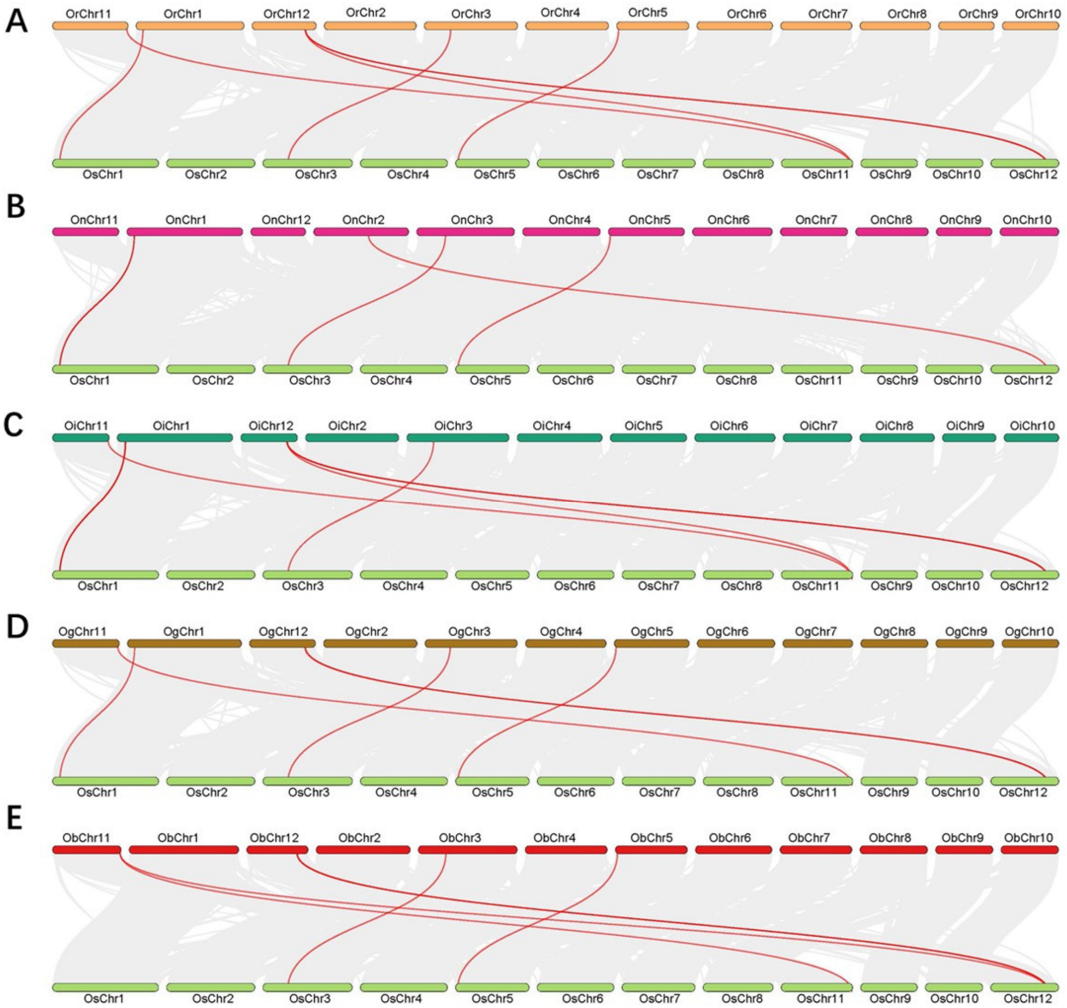

Supplementary Figure S2. Collinearity relationships of *MT* genes in *O. sativa* ssp. *japonica* and O. rufipogon (A), *Oryza nivara* (B), *Oryza sativa* ssp. *indica* (C), *Oryza glumaepatula* (D), *Oryza barthii* (E)

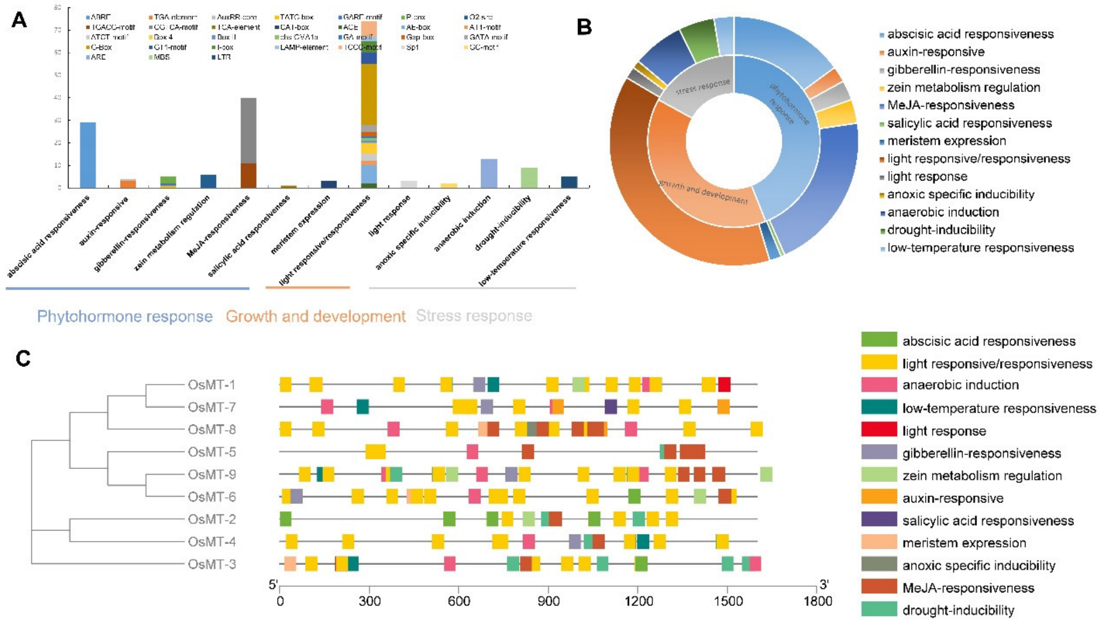

Supplementary Figure S3. Identification of cis-regulatory elements in all *MT* genes of *Oryza sativa* ssp. *japonica*. (A) The different bars represent different primary categories; different characters represent different secondary categories; different colors in the histogram represent the number of different promoter elements in each secondary category. (B) Pie charts of different sizes indicate the ratio of each primary/secondary category. (C) Cis-regulatory elements of all *OsMT* genes in the phylogenetic tree. The differently colored boxes represent the different secondary categories
