# Supplementary material for: Genome-Wide Identification and Analysis of the Metallothionein Genes in Oryza Genus
Source: Int J Mol Sci. 2021 Sep 6;22(17):9651. doi: 10.3390/ijms22179651 (PMC8431808; doi:10.3390/ijms22179651)
Supplement: Supplementary file 1 [file ijms-22-09651-s001.zip › Supplementary/Supplementary Table S1.pdf]

Supplementary Table S1. MT genes were identified in six *Oryza* species/subspecies.

Species	Name Gene	Identifier	CHR	CHR.start	CHR.end	Subfamily
Oryza sativa ssp. japonica	OsMT-1	LOC_Os01g05650.1	Oschr1	2693134	2695085	group 2
	OsMT-2	LOC_Os03g17870.1	Oschr3	9957429	9958013	group 4
	OsMT-3	LOC_Os05g02070.2	Oschr5	605760	606871	group 2
	OsMT-4	LOC_Os11g47809.1	Oschr11	28827676	28828513	group 3
	OsMT-5	LOC_Os12g38010.1	Oschr12	23352191	23353189	group 1
	OsMT-6	LOC_Os12g38051.1	Oschr12	23382984	23391427	group 1
	OsMT-7	LOC_Os12g38270.1	Oschr12	23501361	23502386	group 1
	OsMT-8	LOC_Os12g38290.1	Oschr12	23512497	23513477	group 1
	OsMT-9	LOC_Os12g38300.1	Oschr12	23515147	23515798	group 1
Oryza rufipogon	OrMT-1	ORUFI01G03290.1	Orchr1	2375753	2377195	group 2
	OrMT-2	ORUFI03G13950.1	Orchr3	9631567	9632983	group 4
	OrMT-3	ORUFI11G25490.1	Orchr11	27566267	27574583	group 3
	OrMT-4	ORUFI12G17960.1	Orchr12	19644624	19645305	group 1
	OrMT-5	ORUFI12G17980.1	Orchr12	19654383	19655325	group 1
	OrMT-6	ORUFI12G18000.1	Orchr12	19658884	19661583	group 1
	OrMT-7	ORUFI12G18020.1	Orchr12	19665489	19666156	group 1
	OrMT-8	ORUFI12G18180.1	Orchr12	19787432	19788503	group 1
	OrMT-9	ORUFI12G18190.1	Orchr12	19795680	19798408	group 1
Oryza nivara	OnMT-1	ONIVA01G03530.1	Onchr1	2564823	2566241	group 2
	OnMT-2	ONIVA02G22070.1	Onchr2	20297073	20298088	group 1
	OnMT-3	ONIVA02G22080.4	Onchr2	20309128	20310197	group 1
	OnMT-4	ONIVA03G14560.1	Onchr3	10521390	10523037	group 4
	OnMT-5	ONIVA08G18040.1	Onchr8	19520465	19537852	group 1
	OnMT-6	ONIVA08G18060.1	Onchr8	19542246	19542886	group 1
	OnMT-7	ONIVA10G19390.1	Onchr10	19531399	19532084	group 2
Oryza indica	OiMT-1	BGIOSGA002385-PA	Oichr1	3217847	3218618	group 2
	OiMT-2	BGIOSGA002389-PA	Oichr1	3160509	3161953	group 2
	OiMT-3	BGIOSGA012380-PA	Oichr3	10856082	10856666	group 4
	OiMT-4	BGIOSGA019039-PA	Oichr5	610754	611232	group 2
	OiMT-5	BGIOSGA035740-PA	Oichr11	22867298	22867663	group 3
	OiMT-6	BGIOSGA035979-PA	Oichr12	18905862	18906502	group 1
	OiMT-7	BGIOSGA035981-PA	Oichr12	18899292	18899924	group 1
	OiMT-8	BGIOSGA035984-PA	Oichr12	18867275	18867956	group 1
	OiMT-9	BGIOSGA037630-PA	Oichr12	19026871	19027444	group 1
	OiMT-10	BGIOSGA037631-PA	Oichr12	19029456	19029717	group 1
	OiMT-11	BGIOSGA037632-PA	Oichr12	19037320	19037954	group 1
	OiMT-12	BGIOSGA037633-PA	Oichr12	19039881	19040511	group 1

Supplementary Table S1. Cont.

Species	Name Gene	Identifier	CHR	CHR.start	CHR.end	Subfamily
Oryza glumipatula	OgMT-1	OGLUM01G03480.1	Ogchr1	2753761	2755470	group 2
	OgMT-2	OGLUM03G13410.1	Ogchr3	10121823	10123266	group 4
	OgMT-3	OGLUM10G17340.1	Ogchr10	20756174	20756859	group 2
	OgMT-4	OGLUM11G22770.4	Ogchr11	26716197	26717561	group 3
	OgMT-5	OGLUM12G17670.6	Ogchr12	22609998	22618164	group 1
	OgMT-6	OGLUM12G17910.1	Ogchr12	22834106	22836563	group 1
	OgMT-7	OGLUM12G17920.1	Ogchr12	22840323	22843866	group 1
Oryza barthii	ObMT-1	OBART03G13300.1	Obchr3	9198490	9199770	group 4
	ObMT-2	OBART10G17320.1	Obchr10	17462327	17463010	group 2
	ObMT-3	OBART11G23600.1	Obchr11	22854740	22856090	group 3
	ObMT-4	OBART12G16160.1	Obchr12	16957905	16958586	group 1
	ObMT-5	OBART12G16170.1	Obchr12	16969402	16970344	group 1
	ObMT-6	OBART12G16180.1	Obchr12	16973950	16976647	group 1
	ObMT-7	OBART12G16200.1	Obchr12	16982500	16983167	group 1
	ObMT-8	OBART12G16350.1	Obchr12	17086220	17087191	group 1
	ObMT-9	OBART12G16360.4	Obchr12	17091194	17092245	group 1

Note: CHR/chr in Supplementary Table S1 represents a chromosome. Os represents *Oryza sativa* ssp. *japonica*. Or represents *Oryza rufipogon*. On represents *Oryza nivara*. Oi represents *Oryza sativa* ssp. *indica*. Og represents *Oryza glumaepatula*. Ob represents *Oryza barthii*.
