# Supplementary material for: Genome-Wide Identification and Analysis of the Metallothionein Genes in Oryza Genus
Source: Int J Mol Sci. 2021 Sep 6;22(17):9651. doi: 10.3390/ijms22179651 (PMC8431808; doi:10.3390/ijms22179651)
Supplement: Supplementary file 1 [file ijms-22-09651-s001.zip › Supplementary/Supplementary Table S2.pdf]

Supplementary Table S2. The conserved motifs of *OsMT* genes

Motif ID	Sequence	Width
1	KMYPDLAEKITTTTTTATTVL	21
2	EGVGKAAESGEAAHGCSCGSSCK	23
3	MSCGGSCNCGS	11
4	CNPCNC	6
5	VAPEKGHF	8
6	IITLLSPFLRVKTLFRFSDGRCLRFNAIFLLGDFVLKTLPY	41
7	LFIRSRSCSFELGNDDLCFNLQLYRCKFLLSISHFIQLHKF	41
8	CGCGGGCG	8
9	DDKCGCAVPCPGGTGCRYGDLVALGILATVKPTEQFKCASSARSGGGDHT	50
10	LKSLQETQKIK	11
11	RESQPTGRENRRA	13
12	PAKEQF	6
13	MSTLVGVVFLL	11
14	KMYPDLPEKNTT	12
15	RWRAAD	6
16	PSKGHA	6
17	APEKKA	6
18	QLNLARLP	8
19	STTTTAPAATT	11
20	LEFYNYKF	8
