# Supplementary material for: Genome-Wide Identification and Analysis of the Metallothionein Genes in Oryza Genus
Source: Int J Mol Sci. 2021 Sep 6;22(17):9651. doi: 10.3390/ijms22179651 (PMC8431808; doi:10.3390/ijms22179651)
Supplement: Supplementary file 1 [file ijms-22-09651-s001.zip › Supplementary/Supplementary Table S3.pdf]

Supplementary Table S3. the MT genes in each orthogroup

Name	Os	Or	On	Oi	Og	Ob
Orthogroup0	OsMT-6	OrMT-7, OrMT-9	OnMT-6	OiMT-6, OiMT-7, OiMT-12	OgMT-5, OgMT-7	ObMT-7
Orthogroup1	OsMT-2	OrMT-2	OnMT-4	OiMT-3	OgMT-2	ObMT-1
Orthogroup2	OsMT-4	OrMT-3		OiMT-5, OiMT-10	OgMT-4	ObMT-3
Orthogroup3	OsMT-8	OrMT-5	OnMT-3	OiMT-11		ObMT-5, ObMT-9
Orthogroup4	OsMT-7	OrMT-8	OnMT-2	OiMT-9	OgMT-6	ObMT-8
Orthogroup5	OsMT-1	OrMT-1	OnMT-1	OiMT-1, OiMT-2	OgMT-1	
Orthogroup6	OsMT-5, OsMT-9	OrMT-4		OiMT-8		ObMT-4
Orthogroup7			OnMT-7		OgMT-3	ObMT-2
Orthogroup8		OrMT-6	OnMT-5			ObMT-6
