# Supplementary material for: Genome-Wide Identification and Analysis of the Metallothionein Genes in Oryza Genus
Source: Int J Mol Sci. 2021 Sep 6;22(17):9651. doi: 10.3390/ijms22179651 (PMC8431808; doi:10.3390/ijms22179651)
Supplement: Supplementary file 1 [file ijms-22-09651-s001.zip › Supplementary/Supplementary Table S4.pdf]

Supplementary Table S4. Analysis of orthologous groups genes in six *Oryza* Genus

**Name**	**Os**	**Or**	**On**	**Oi**	**Og**	**Ob**	**Total**
Orthogroup0	1	2	1	3	2	1	10
Orthogroup1	1	1	1	1	1	1	6
Orthogroup2	1	1	0	2	1	1	6
Orthogroup3	1	1	1	1	0	2	6
Orthogroup4	1	1	1	1	1	1	6
Orthogroup5	1	1	1	2	1	0	6
Orthogroup6	2	1	0	1	0	1	5
Orthogroup7	0	0	1	0	1	1	3
Orthogroup8	0	0	1	0	1	1	3
