# Supplementary material for: Genome-Wide Identification and Analysis of the Metallothionein Genes in Oryza Genus
Source: Int J Mol Sci. 2021 Sep 6;22(17):9651. doi: 10.3390/ijms22179651 (PMC8431808; doi:10.3390/ijms22179651)
Supplement: Supplementary file 1 [file ijms-22-09651-s001.zip › Supplementary/Supplementary Table S6.pdf]

Supplementary Table S6. List of oligonucleotide primers used for qRT-PCR analysis

Name	Forward (+)	Reverse (-)
OsMT-1QPCR	ATGTCGTGCTGCGGAGGAAA	TCACTTGCCGCAGTTGCAGG
OsMT-2QPCR	CTGTGGATCAAGCTGTGGCT	CTCGAAGTGCGCCTTCTCC
OsMT-3QPCR	CCACCAAGACCTTCGTCCTC	TAAATCCTCATGCAGCCGGG
OsMT-4QPCR	CAAACTGCTCCTGCGGAAAG	TCAAACTGCTGCGCCTTCTT
OsMT-5QPCR	CCATCACCACTACTACTACAACCA	ATGGGCACCCTCACCAGATT
OsMT-6QPCR	AGATGTACCCTGACCTGGCT	GATTCAGCTGCCTTGCCAAC
OsMT-7QPCR	CAAGAAGATGTCTTGCTGCGGA	CCCCGAGGACCATAGTTGCT
OsMT-8QPCR	TAAGCTGCGGCTGCGGAAAG	AGCAGCCTCGAACACCGCTT
OsMT-9QPCR	GTCCTAGGCACTCTGAGGGG	CCTTAGCAGTTGCAGGGGTT
Actin	TGCTATGTACGTCGCCATCCAG	AATGAGTAACCACGCTCCGTCA
