# Supplementary material for: Genome-Wide Identification and Analysis of the Metallothionein Genes in Oryza Genus
Source: Int J Mol Sci. 2021 Sep 6;22(17):9651. doi: 10.3390/ijms22179651 (PMC8431808; doi:10.3390/ijms22179651)
Supplement: Supplementary file 1 [file ijms-22-09651-s001.zip › Supplementary/Supplementary Table S7.pdf]

Supplementary Table S7. List of oligonucleotide primers used for subcellular localization analysis

Name	Forward (+)	Reverse (-)
HBT-OsMT-1	CCTCTCCCCTTGCTCCGTGGATCCATGTCGTGCTGCGGAGGAAACT	CTCGCCCTTGCTCACCATGGATCCCTTGCCGCAGTTGCAGGGGTTGC
HBT-OsMT-3	CCTCTCCCCTTGCTCCGTGGATCCATGGATGCAGATGCAAGATG	CTCGCCCTTGCTCACCATGGATCCAAGCTCTGATCGACAGTAGC
HBT-OsMT-4	CCTCTCCCCTTGCTCCGTGGATCCATGTCTTGCAGCTGTGGATCTA	CTCGCCCTTGCTCACCATGGATCCGCAGTTGCAAGGGTTGCACCTG
HBT-OsMT-5	CCTCTCCCCTTGCTCCGTGGATCCATGTCGTGCGGTGGAAGTT-GCAACT	CTCGCCCTTGCTCACCATGGATCCGCAGTTGCAGGGGTTGCACTT-GCAG
HBT-OsMT-6	CCTCTCCCCTTGCTCCGTGGATCCATGTCGTGCGGTGGAAGTTGCAA	CTCGCCCTTGCTCACCATGGATCCGCAGTTGCAGGGGTTGCACTTGC
HBT-OsMT-7	CCTCTCCCCTTGCTCCGTGGATCCATGCATTGCACTCATCTCAA-GCTTTT	CTCGCCCTTGCTCACCATGGATCCGCAGTTGCAGGGATTGCATTTGCA
