# Supplementary material for: Genome-Wide Identification and Analysis of the Metallothionein Genes in Oryza Genus
Source: Int J Mol Sci. 2021 Sep 6;22(17):9651. doi: 10.3390/ijms22179651 (PMC8431808; doi:10.3390/ijms22179651)
Supplement: Supplementary file 1 [file ijms-22-09651-s001.zip › Supplementary/Supplementary Table S8.pdf]

Supplementary Table S8. List of oligonucleotide primers used for dual-luciferase assays

Name	Forward (+)	Reverse (-)
OsMT-1Pro	TCGAATTCCTGCAGCCCGGGGGATCCcttttgaacacgacgtttaaccgtcca	GCGGCCGCTCTAGAACTAGTGGATCCatcgatcgaactgcgggcactga
OsMT-2Pro	TCGAATTCCTGCAGCCCGGGGGATCCtttggagacgtgtcacaaatttcca	GCGGCCGCTCTAGAACTAGTGGATCCtgacaaggagatgaagggctttgaga
OsMT-3Pro	TCGAATTCCTGCAGCCCGGGGGATCCagctagagtccaacacggtcgaaaa	GCGGCCGCTCTAGAACTAGTGGATCCctgtgcgtgtcacaatggtttcttg
OsMT-4Pro	TCGAATTCCTGCAGCCCGGGGGATCCattgcagtgagtaccacccatggt	GCGGCCGCTCTAGAACTAGTGGATCCgcaatgcatagccttcgggtcctcc
OsMT-5Pro	TCGAATTCCTGCAGCCCGGGGGATCCtgttgctatactagtgtgatttggtc	GCGGCCGCTCTAGAACTAGTGGATCCgatgtagctggttgaaatggccttg
OsMT-6Pro	TCGAATTCCTGCAGCCCGGGGGATCCgaagctacggctgttattccatgta	GCGGCCGCTCTAGAACTAGTGGATCCcttcaggaacttgtatgcaatgagag
OsMT-7Pro	TCGAATTCCTGCAGCCCGGGGGATCCatgtcaattttgaaacgagcctttgc	GCGGCCGCTCTAGAACTAGTGGATCCattgctgaaagcataccatgatcctata
OsMT-8Pro	TCGAATTCCTGCAGCCCGGGGGATCCgcacacgacctgacttggcatttg	GCGGCCGCTCTAGAACTAGTGGATCCtccatcttgcttcaatactatgatgctga
OsMT-9Pro	TCGAATTCCTGCAGCCCGGGGGATCCttcccattgcttggtcagatggctg	GCGGCCGCTCTAGAACTAGTGGATCCcttgatcgtctgggtctcttgtaaact
